# Highlight: Strange Bedfellows: The Origin and Evolution of Bacterial Hybrids

**DOI:** 10.1093/gbe/evac146

**Published:** 2022-10-10

**Authors:** Casey McGrath

The creation of an interspecies hybrid—an organism containing genes from more than one species—is an extremely common occurrence in the realm of bacteria. The transfer of genetic material from one bacterial species to another is often referred to as lateral or horizontal transfer and plays a major role in bacterial evolution and diversity, for example in the exchange of antibiotic resistance genes among bacteria. Horizontal genetic transfer can occur through several different mechanisms, some of which are still poorly understood. In some cases, a stretch of DNA in the recipient bacterium is replaced with a similar stretch of DNA from the donor bacterium, leaving the overall size of the bacterial chromosome unchanged. In contrast, some bacterial hybridization events increase the length of the bacterial chromosome. In a new study in *Genome Biology and Evolution*, Professor Katrin Bartke et al. at Uppsala University provide insight into the mechanisms that create bacterial hybrids with enlarged chromosomes and their subsequent evolutionary trajectories (Bartke et al. 2022).

Bartke et al. have “been interested for some time in the genetic and evolutionary aspects of chromosome dynamics in bacteria. Bacteria live naturally in environments with other species so that there is almost always the potential for interaction and transfer of DNA from one to another.” Indeed, the authors note the recent discovery of two globally widespread hybrid bacterial pathogens (*Klebsiella pneumoniae* ST258 and *Escherichia coli* ST1193), each with at least 20% of their chromosome originating from a separate species, indicating the potential importance of bacterial hybrids, both clinically and evolutionarily.

To better understand this process, the authors mixed together two bacterial strains in the lab that have been evolving as separate species for over 100 million years: *Salmonella enterica* serovar Typhimurium and *E. coli* Hfr, a strain known to engage in horizontal transfer at high frequencies. The Hfr strain of *E. coli* has the ability to act as a donor cell, transferring some of its genetic material to a recipient cell. The authors then selected for hybrid cells carrying markers for both parental strains and identified eight strains in which *E. coli* DNA had recombined into the *Salmonella* chromosome, resulting in an increase in chromosome length.

**Fig. 1.**
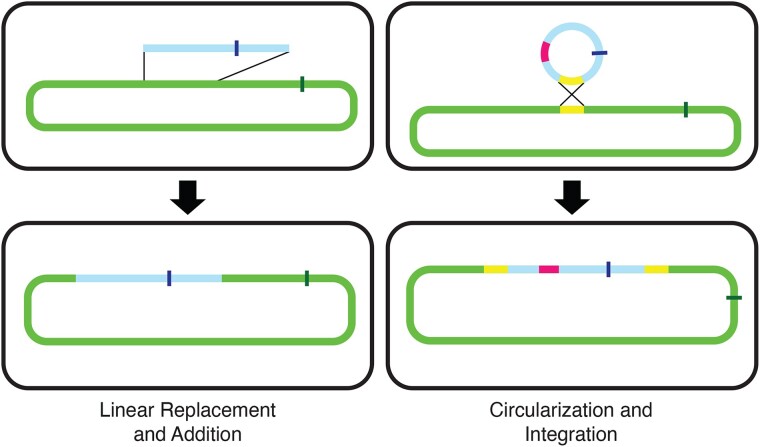
Different mechanisms by which chromosome size can be enlarged in hybrids. Linear replacement and addition: A linear fragment of donor DNA is transferred into a recipient, where it recombines into the recipient chromosome and replaces a smaller section of recipient DNA. Circularization and Integration: The linear donor DNA recombines into a circular molecule, which then recombines into the recipient chromosome, resulting in chromosome enlargement without the loss of any recipient DNA.

In some cases, these increases were substantial, ranging from 151 kb to 1.3 Mb. The authors were surprised “that such huge fragments of DNA—in one case a quarter of the length of the donor chromosome—could be recombined into the recipient chromosome.” Moreover, these enlarged chromosomes resulted from multiple distinct mechanisms (fig. 1). In five cases, a stretch of *Salmonella* DNA was replaced with a larger length of *E. coli* DNA, while in the other three cases, a circular fragment of *E. coli* DNA recombined into the *Salmonella* chromosome, thus increasing the length of the chromosome without the loss of any *Salmonella* DNA. This latter mechanism is particularly intriguing, as in principle it could occur between even distantly related species since it does not require high levels of similarity between donor and recipient genomes, either in terms of nucleotide sequence or gene order.

Among the eight hybrid strains identified by Bartke et al., all but one exhibited a reduced growth rate compared to the parental *Salmonella* strain, indicating a negative effect on fitness, at least under lab conditions. To investigate the evolutionary trajectories of these hybrids, the authors performed experimental evolution of two of the hybrid strains, evolving five independent lineages for each strain for 100 generations under two conditions: with and without selection for the antibiotic resistance marker that delineated the *E. coli* DNA fragment.

All of the lineages increased in relative growth rate, in some cases achieving a growth rate on par with the parental *Salmonella* strain. Perhaps not surprisingly, in most cases, this was associated with chromosomal deletions that included part of the integrated *E. coli* DNA fragment. What was surprising, however, is that the authors never observed the precise deletion of the entire *E. coli* segment. Instead, in all cases, the deletions included both acquired *E. coli* and resident *Salmonella* DNA, leaving irreversibly hybrid chromosomes. As noted by the authors, this outcome was “strongly influenced by the interplay between the selection pressure for increased growth fitness and the chromosomal distribution of sequences that can act as substrates for deletion by homologous recombination.”

Based on these results, Bartke et al. believe that “the formation of hybrid chromosomes is probably a common event in natural environments, but detection might be easily obscured, for example by subsequent evolution.” However, they recognize that additional work is needed to confirm this. “One potential criticism is that we are looking at a laboratory phenomenon and that we have not shown evidence for similar events occurring in natural isolates or environments.” The authors are currently working to address this criticism. “We are interested in examining these phenomena both experimentally and using bioinformatic approaches. Using controlled laboratory approaches, we can become familiar with the potential genetic signatures of hybrid chromosomes, and this might make it easier to recognize them in natural isolates.”

Bartke et al. believes that these efforts will eventually be successful: “While our experiments are done in the lab, analogous experiments are being done constantly in the environment because of the vast number of bacteria that exist and are constantly interacting…Given the large population size of bacteria on Earth, it is very likely that similar events to those we have observed in our experiments also occur naturally.”
